# Additional diagnostic value of cervical ultrasound in the detection of cervical lymph node metastases in patients with esophageal cancer

**DOI:** 10.1093/dote/doaf135

**Published:** 2026-01-22

**Authors:** Jasmijn R van Doesburg, Nannet Schuring, Mark H M Vries, Pim de Graaf, Katya M Duvivier, Freek Daams, Mark I van Berge Henegouwen, Suzanne S Gisbertz

**Affiliations:** Department of Surgery, Amsterdam UMC location Vrije Universiteit, Amsterdam, Noord-Holland, The Netherlands; Department of Surgery, Amsterdam UMC location University of Amsterdam, Amsterdam, Noord-Holland, The Netherlands; Cancer Treatment and Quality of Life, Cancer Center Amsterdam, Amsterdam, Noord-Holland, The Netherlands; Amsterdam Gastroenterology Endocrinology Metabolism Research Institute, Amsterdam, Noord-Holland, The Netherlands; Department of Surgery, Amsterdam UMC location Vrije Universiteit, Amsterdam, Noord-Holland, The Netherlands; Department of Surgery, Amsterdam UMC location University of Amsterdam, Amsterdam, Noord-Holland, The Netherlands; Department of Radiology, Amsterdam UMC location Vrije Universiteit, Amsterdam, Noord-Holland, The Netherlands; Department of Radiology, Amsterdam UMC location Vrije Universiteit, Amsterdam, Noord-Holland, The Netherlands; Cancer Center Amsterdam, Imaging and Biomarkers, Amsterdam, Noord-Holland, The Netherlands; Department of Radiology, Amsterdam UMC location Vrije Universiteit, Amsterdam, Noord-Holland, The Netherlands; Department of Surgery, Amsterdam UMC location Vrije Universiteit, Amsterdam, Noord-Holland, The Netherlands; Cancer Treatment and Quality of Life, Cancer Center Amsterdam, Amsterdam, Noord-Holland, The Netherlands; Department of Surgery, Amsterdam UMC location University of Amsterdam, Amsterdam, Noord-Holland, The Netherlands; Cancer Treatment and Quality of Life, Cancer Center Amsterdam, Amsterdam, Noord-Holland, The Netherlands; Amsterdam Gastroenterology Endocrinology Metabolism Research Institute, Amsterdam, Noord-Holland, The Netherlands; Department of Surgery, Amsterdam UMC location University of Amsterdam, Amsterdam, Noord-Holland, The Netherlands; Cancer Treatment and Quality of Life, Cancer Center Amsterdam, Amsterdam, Noord-Holland, The Netherlands

**Keywords:** cancer staging, cervical ultrasound (US), computer tomography (CT), esophageal cancer, positron emission tomography (PET), ultrasound-guided fine needle aspiration cytology (USGFNAC)

## Abstract

In Western Europe, esophageal cancer patients with cervical lymph node metastases are considered to have stage IV disease and are generally not eligible for curative treatment. While cervical ultrasound was part of standard diagnostic workup, its added value after negative ^18^FDG PET-CT is debated, and ultrasound is no longer in the Dutch guideline as standard workup modality. This study assessed the diagnostic accuracy of ultrasound for the detection of cervical lymph node metastases in esophageal cancer patients. This retrospective cohort study included all esophageal cancer patients referred to or diagnosed at the Amsterdam UMC between January 2014 and January 2021. Radiology and multidisciplinary team meeting reports were reviewed to identify patients with suspicious cervical lymph node(s). Primary outcome was the detection rate of cervical lymph node metastases on ultrasound and/or ^18^FDG PET-CT. The gold standard was fine needle aspiration. This study included 747 patients; median age was 67 years. Patients were predominantly male (75.5%) and majority had an adenocarcinoma (72.0%). Total of 112 (15.0%) patients had suspicious cervical lymph nodes, with malignancy confirmed in 38 cases. Cervical ultrasound showed high sensitivity (94.7%), but low positive predictive value (37.1%) compared to ^18^FDG PET-CT, which had 100% sensitivity, 91.3% specificity, and 71.7% PPV. This study demonstrated that cervical ultrasound offers no additional diagnostic value over ^18^FDG PET-CT alone in the assessment of cervical lymph node metastases during diagnostic workup for esophageal cancer and increases the number of fine needle aspirations conducted for benign cervical lymph nodes.

## INTRODUCTION

After curative treatment, usually consisting of neoadjuvant chemoradiotherapy and esophagectomy with lymphadenectomy, the current 5-year survival rate of esophageal cancer is 47%.^1^ Whether or not a patient can be treated with curative intent depends on several tumor-specific characteristics, i.e. the clinical stage of the disease and patient-specific characteristics, such as physical fitness. Esophageal cancer patients with stage IV disease, according to the 8th edition TNM clinical classification system, are generally not eligible for treatment with curative intent.^2^ According to the 8th edition of the American Joint Committee on Cancer (AJCC), cervical metastases, apart from station 1, level VI, and level VII, are regarded as distant metastases.^3^ Currently, the clinical imaging techniques used in the diagnostic workup of esophageal cancer patients consist of 18F-fluorodeoxyglucose (^18^FDG) positron emission tomography (PET) with integrated diagnostic computed tomography (CT). On indication, endoscopic ultrasound (EUS) and cervical ultrasound with or without fine needle aspiration (FNA) are performed.^4^^,^^5^ Until recently, cervical ultrasound was part of the routine diagnostic workup for esophageal cancer. However, since the development of integrated ^18^FDG PET-CT systems has led to more accurate imaging of cervical lymph nodes, the additional value of cervical ultrasound has been disputed. Few studies investigated the additional value of neck ultrasound in the diagnostic workup of esophageal cancer after a negative ^18^FDG PET-CT for the detection of cervical lymph node metastases.^6–11^ Results were heterogeneous, and none of the current studies were conducted in larger cohorts. Since previous evidence is limited and has conflicting results and the identification of cervical metastases has major consequences, this study aimed to assess the sensitivity and specificity of cervical ultrasound for the detection of malignant cervical lymph nodes in patients with esophageal cancer, to evaluate the additional value of cervical ultrasound over negative ^18^FDG PET-CT. The secondary aim of this study was to assess the proportion of patients who unnecessarily undergo an invasive procedure due to a false-positive finding on cervical ultrasound after a negative ^18^FDG PET-CT.

## METHODS

### Study design

In this retrospective cohort study, all cervical ultrasound and ^18^FDG PET-CT imaging reports and all multidisciplinary team meeting reports established during the esophageal cancer diagnostic workup were manually reviewed for the presence of cervical lymph nodes suspected of metastases. Informed consent was waived by the local medical ethics commission (W19_097#19.127). This study was reported according to the Strengthening the Reporting of Observational Studies in Epidemiology (STROBE) reporting checklist and the Standards for Reporting of Diagnostic Accuracy guideline.^12^^,^^13^

### Setting and study population

**Fig. 1:**
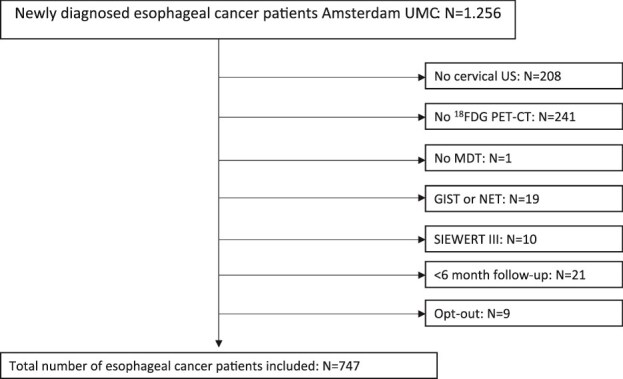
Flow chart of patient selection.

All esophageal cancer patients, including gastro-esophageal junction cancer patients, referred to or diagnosed in the Amsterdam UMC between January 1, 2014, and January 1, 2021, were considered for inclusion. Patients were excluded when they met one of the following criteria: (1) No cervical ultrasound was performed during the diagnostic workup, or images and/or study report were not available. (2) No ^18^FDG PET-CT was performed during the diagnostic workup, or images and/or study report were not available. (3) The patient was not discussed in an multidisciplinary (MDT) meeting. (4) Tumor histology comprised a gastrointestinal stromal tumor or a neuroendocrine tumor. (5) Siewert type III tumor. (6) Patients with less than 6 months of follow-up. (7) Patient applied for opt-out. The flowchart of patient selection is displayed in Fig. 1.

### Staging, treatment, and follow-up

Esophageal cancer staging was performed according to the 8th edition of the AJCC TNM cancer staging^2^ and was generally performed using endoscopy with biopsies, ultrasound of the neck, and ^18^FDG PET-CT. On indication, endoscopic ultrasound was performed (e.g. suspected T4b tumor or lymph node metastases outside the planned radiation field or surgical resection field). Patients eligible for curative treatment generally underwent neo-adjuvant chemoradiotherapy according to the Chemoradiotherapy for Oesophageal Cancer followed by Surgery Study (CROSS) scheme, followed by an esophagectomy.^14^ After discharge, patients were followed up at the outpatient clinic. In the first postoperative year, follow-up visits were scheduled every 3 months. In the second to fourth postoperative year, follow-up was scheduled every 6 months. Thereafter, patients visited the outpatient clinic once more in the fifth postoperative year.^5^ In case a recurrence of esophageal cancer was suspected, patients underwent an ^18^FDG PET-CT, CT scan of the neck, thorax and abdomen, or endoscopy. Patients with potentially curable esophageal cancer ineligible for surgery received definitive chemoradiotherapy. Patients who were not found eligible for curative treatment received palliative treatment or best supportive care. The follow-up procedure was not standardized for these patients.

### Test methods, data source, and variables

Data on patient and tumor characteristics were obtained from a prospectively maintained database, and missing data were extracted from clinical files. Lymph nodes were classified as locoregional or distant metastases according to the American Joint Committee on Cancer (AJCC) manual.^3^ Thus, lymph nodes in station 1 L/R, level VI, and VII were considered locoregional. In accordance with Dutch guidelines, a suspicious lymph node is defined as a node >9 mm short axis or as a node 5–9 mm short axis that is round, inhomogeneous, and ill-defined. Other suspicious characteristics can comprise FDG-positivity on ^18^FDG PET/CT or a clear asymmetry in size compared with the contralateral neck.^5^^,^^15–17^ Fine needle aspiration was used as the gold standard. Patients with a suspicious lymph node with uncertain etiology, as no conclusive pathology was available, were excluded when calculating diagnostic accuracy measures. To guarantee data accuracy, all patients with a suspicious cervical lymph node for which there was any uncertainty about the lymph node level, size, FDG avidity, or other discrepancy, ^18^FDG PET/CT and/or ultrasound imaging were retrospectively reviewed by an experienced radiologist.

### Statistics

Descriptive statistics were generated based on all subjects in the analytical cohort. Normality was tested using histograms. In case of a skewed distribution, medians with interquartile ranges (IQRs) were reported. Categorical data were shown in tables as absolute numbers with associated fractions (%). When calculating proportions, missing values were not counted. Comparisons of patient and tumor characteristics among those with benign and malignant cervical lymph nodes were performed using Pearson χ^2^ for nominal data and Mann–Whitney *U* tests for nonnormally distributed continuous data. A *P*-value of less than 0.05 was considered statistically significant. All statistical tests were two-sided. Sensitivity, specificity, positive predictive value (PPV), and negative predictive value (NPV) for ultrasound and ^18^FDG PET-CT were calculated. Data were stored and analyzed using IBM SPSS Statistics for Windows, Version 26.0. Armonk, NY: IBM Corp Released 2019.

## RESULTS

### Population

A total of 747 esophageal cancer patients were included in this study. Median age of patients was 67 years, and the majority had an adenocarcinoma (72.0%). In this cohort, a cT3 tumor was most frequently diagnosed (69.5%). Most patients had one or two suspected regional lymph node metastases upon diagnosis (cN1; 39.1%) and no distant metastasis (cM0; 76.4%). Table 1 shows the baseline characteristics of all included patients.

**Table 1:** Baseline characteristics of all included patients and patients with suspicious cervical lymph nodes

		All patients *N* = 747		Suspicious cervical lymph nodes *N* = 112		Malignant *N* = 38	
		*N*	%	*N*	%	*N*	%
**Sex**	Male	564	75.5	84	75.0	30	78.9
	Female	183	24.5	37	25.0	8	20.1
**Age at diagnosis**	Years, median (IQR)	67.0 (60.0–73.0)		65.5 (58.0.72.0)		63.0 (57.8–72.0)	
Comorbidity index score^*^	0–3	488	65.3	77	68.8	29	76.3
	4–6	233	31.2	33	29.5	9	23.7
	≥7	26	3.5	2	1.8	—	—
	Median (IQR)	3.0 (2.0–4.0)		3.0 (2.0–4.0)		2.5 (1.0–3.3)	
**ASA-class**	ASA 1	56	10.1	5	7.0	1	8.3
	ASA 2	320	57.8	42	59.2	8	66.7
	ASA 3	171	30.9	22	31.0	1	8.3
	ASA 4	7	1.3	2	2.8	2	16.7
**Histology**	Adenocarcinoma	538	72.0	65	58.0	17	44.7
	Squamous cell carcinoma	208	27.8	47	42.0	21	55.3
	Adenosquamous carcinoma	1	0.1	—	—	—	—
**Primary tumor location**	Proximal	39	5.2	10	8.9	5	13.2
	Mid	136	18.2	30	26.8	14	36.8
	Distal/GEJ	445	59.6	72	64.3	19	50.0
	Multifocal	6	0.8	—	—	—	—
**Treatment type**	Neoadjuvant + resection	412	55.5	46	41.1	—	—
	Resection	22	3.0	3	2.8	—	—
	dCRT	166	22.3	32	28.6	17	44.7
	Palliative	134	18.0	29	25.9	21	55.3
	Best supportive care	6	0.8	2	1.8	—	—
	Endoscopic resection	3	0.4	—	—	—	—
**cT**	T1	26	3.5	3	2.7	1	2.7
	T2	115	15.5	14	12.6	1	2.7
	T3	514	69.5	73	65.8	21	56.8
	T4	53	7.2	13	11.7	8	21.6
	Tx	32	4.3	8	7.2	6	16.2
**cN**	N0	206	27.8	20	18.0	—	—
	N1	290	39.1	48	43.2	9	24.3
	N2	184	24.8	29	26.1	17	45.9
	N3	44	5.9	12	10.8	10	27.0
	N+	5	0.7	1	0.9	1	2.7
	Nx	12	1.6	1	0.9	—	—
**cM**	M0	569	76.4	59	52.7	—	—
	M1	100	13.4	42	37.5	38	100
	Mx	76	10.2	11	9.8	—	—

IQR, interquartile range; ASA-class, American Society of Anesthesiologists Physical Status Classification System; GEJ, gastro-esophageal junction; dCRT, definitive chemoradiotherapy; cT, clinical primary tumor stage; cN, clinical regional lymph nodes stage; cM, clinical distant metastasis stage, TNM classification of malignant tumours, 8th edition. An em dash (—) was noted when there were no patients with these characteristics.

^*^Weighting as contemplated in the Charlson Comorbidity index.^10^

### Cervical lymphadenopathy

Of all 747 patients, 112 (15.0%) had one or more cervical lymph node(s) that were suspect for metastases based on ^18^FDG PET-CT and/or ultrasound. Fine needle aspiration was performed in 109 out of 112 patients (97.3%). Of those, 38 proved to have one or more malignant lymph nodes and 65 benign. For nine patients (8.0%), etiology of the suspicious lymph node(s) remained uncertain, as there was no pathology available or fine needle aspiration was inconclusive and no further investigations were performed, as this was not deemed clinically relevant. No major complications after FNA were reported. Table 2 summarizes lymph node characteristics of all patients with a suspicious cervical lymph node and the modality upon which these were diagnosed. Of the 38 proven malignant lymph nodes, two (5.3%) were detected with ^18^FDG PET-CT and not visualized on screening ultrasound during the diagnostic workup. None of the malignant lymph nodes were detected by ultrasound alone, with a negative ^18^FDG PET-CT.

**Table 2:** Lymph node characteristics and modality

		Suspicious cervical lymph nodes *N* = 112		Benign cervical lymph node *N* = 65		Malignant cervical lymph node *N* = 38		Lymph node with unclear etiology *N* = 9	
		*N*	%	*N*	%	*N*	%	*N*	%
**Modality**	^18^FDG-PET-CT	8	7.1	4	16.9	2	5.3	2	22.2
	US	54	48.2	50	76.9	—	—	4	44.4
	Both	50	44.6	11	16.9	36	94.7	3	33.3
**FDG avidity** ^*^	No	62	55.4	54	83.1	3	7.9	5	55.6
	Yes	50	44.6	11	16.9	35	92.1	4	44.4
**Laterality**	Left	50	44.6	31	47.7	17	44.7	2	22.2
	Right	41	36.6	24	36.9	12	31.6	5	55.6
	Both	21	18.8	10	15.4	9	23.7	2	22.2
**Number of suspicious nodes**	1	76	68.5	49	75.4	22	59.5	5	55.6
	≥2	36	32.1	16	24.6	16	42.1	4	44.4
**Level** ^*^	I	7	6.3	7	10.9	—	—	—	—
	II	23	20.7	18	28.1	3	7.9	2	22.2
	III	13	11.7	8	12.5	2	5.3	3	33.3
	IV	65	58.6	30	46.9	32	84.2	3	33.3
	V	3	2.7	1	1.6	1	2.6	1	11.1
**Size (mm)** ^*^	Median (IQR)	6.0 (5.0–9.0)		5.0 (4.5–7.0)		11.0 (7.8–15.3)		7.0 (5.0–8.8)	
**FNA**	Yes	109	97.3	65	100	38	100	6	66.7
	No	3	2.7	—	—	—	—	3	33.3

^18^FDG-PET, fluorodeoxyglucose-positron emission tomography; CT, computed tomography; US, cervical ultrasound; FDG, fluorodeoxyglucose; IQR, interquartile range. FNA, fine needle aspiration. An em dash (—) was noted when there were no patients with these characteristics.

^*^In case a patient had multiple suspicious lymph nodes, the largest was analyzed.

### Patient characteristics by lymph node status

Table 3 shows the baseline characteristics of all patients with a suspicious cervical lymph node, comparing patients with benign versus malignant cervical lymph node involvement. No significant differences were observed between the two groups regarding sex (*P* = 0.454), age at diagnosis (*P* = 0.318), or comorbidity index score (*P* = 0.323). However, histological analysis revealed a significantly higher incidence of squamous cell carcinoma (SCC) in the malignant group (55.5% vs. 32.3%, *P* = 0.022), while adenocarcinoma was more common in the benign group. Primary tumor location also differed significantly between groups, with tumors in the distal esophagus or gastroesophageal junction (GEJ) more often associated with benign cervical nodes (73.8% vs. 50.0%, *P* = 0.040). Patients with malignant cervical lymph nodes more frequently presented with higher cT classifications (cT3: 47.2% vs. 15.6%; cT4: 25.8% vs. 7.9%), although this difference did not reach statistical significance (*P* = 0.052). Additionally, cN classifications did show significant differences. Patients with malignant nodes had more nodal involvement (*P* < 0.001).

**Table 3:** Baseline characteristics of suspicious cervical lymph nodes, benign versus malignant

		Benign cervical lymph nodes *N* = 65		Malignant cervical lymph nodes *N* = 38		*P*-value
		*N*	%	*N*	%	
**Sex**	Male	47	72.3	30	78.9	
	Female	18	27.7	8	21.1	0.454^a^
**Age at diagnosis**	Years, median (IQR)	66 (59.5–73.0)		63.0 (57.8–72.0)		0.318^b^
Comorbidity index score^*^	0–3	42	64.6	29	76.3	
	4–6	21	32.3	9	23.7	
	≥7	2	3.1	—	—	0.323^a^
	Median (IQR)	3.0 (2.0–4.0)		2.5 (1.0–3.3)		0.145^b^
**ASA-class**	ASA 1	4	7.4	1	8.3	
	ASA 2	33	61.1	8	66.7	
	ASA 3	17	31.5	1	8.3	
	ASA 4	—	—	2	16.7	0.012^a^
**Histology**	Adenocarcinoma	44	67.7	17	44.7	
	SCC	21	32.3	21	55.5	0.022^a^
**Primary tumor location**	Proximal	3	4.6	5	13.2	
	Mid	14	21.5	14	36.8	
	Distal/GEJ	48	73.8	19	50.0	0.040^a^
**Treatment type**	(neoadjuvant) + resection	46	70.8	—	—	
	dCRT	11	16.9	17	44.7	
	Palliative/BSC	8	12.3	21	55.3	<0.001^a^
**cT**	T1	2	3.2	1	3.2	
	T2	10	15.9	1	3.2	
	T3	46	73.0	21	67.7	
	T4	5	7.9	8	25.8	0.052^a^
**cN**	N0	16	25.0	—	—	
	N1	36	56.3	9	25.0	
	N2	10	15.6	17	47.2	
	N3	2	3.1	10	27.8	<0.001^a^
**cM**	M0	53	81.5	—	—	
	M1	3	4.6	38	100	
	Mx	9	13.8	—	—	

IQR, interquartile range; ASA-class, American Society of Anesthesiologists Physical Status Classification System; GEJ, gastro-esophageal junction; SCC, squamous cell carcinoma; dCRT, definitive chemoradiotherapy; BSC, Best Supportive Care; cT, clinical primary tumor stage; cN, clinical regional lymph nodes stage; cM, clinical distant metastasis stage, TNM classification of malignant tumours, 8th edition. An em dash (—) was noted when there were no patients with these characteristics.

^*^Weighting as contemplated in the Charlson Comorbidity index.^10^

^a^ *P*-value was calculated using a two-sided χ^2^ test.

^b^ *P*-value was calculated using Mann–Whitney *U* test.

### Cervical lymph node characteristics by lymph node status

Table 4 illustrates the lymph node characteristics of benign versus malignant cervical lymph nodes. Most benign cervical lymph showed no FDG avidity (83.1%), whereas most malignant cervical lymph nodes did (92.1%). This difference was found to be statistically significant (*P* < 0.001). Patients with a malignant cervical lymph node often presented with more than one suspicious cervical lymph node (40.5%) compared to the benign group (24.6%), though this did not reach statistical significance (*P* = 0.064). Malignant cervical lymph nodes were significantly larger than benign cervical lymph nodes, with a median size of 11 and 5.0 mm, respectively (*P* < 0.001). Additionally, a statistically significant difference (*P* = 0.003) was observed between the level at which suspicious lymph nodes were detected in benign versus malignant cases. Malignant lymph nodes were predominantly found at level IV, with an occurrence rate of 84.2%, compared to 46.9% in benign cases.

**Table 4:** Benign versus malignant lymph node characteristics

		Benign cervical lymph nodes *N* = 65		Malignant cervical lymph nodes *N* = 38		*P*-value
		***N***	**%**	***N***	**%**	
**Modality**	^18^FDG PET-CT	4	16.9	2	5.3	
	US	50	76.9	—	—	
	Both	11	16.9	36	94.7	<0.001^a^
**FDG avidity** ^*^	No	54	83.1	3	7.9	
	Yes	11	16.9	35	92.1	<0.001^a^
**Laterality**	Left	31	47.7	17	44.7	
	Right	24	36.9	12	31.6	
	Both	10	15.4	9	23.7	0.567^a^
**Number of suspicious nodes**	1	49	75.4	22	59.5	
	≥2	16	24.6	16	42.1	0.064^a^
**Level** ^*^	I	7	10.9	—	—	
	II	17	26.6	3	7.9	
	III	8	12.5	—	—	
	IV	30	46.9	32	84.2	
	V	1	1.6	1	2.6	0.003^a^
**Size (mm)** ^*^	Median (IQR)	5.0 (4.5–7.0)		11.0 (7.8–15.3)		<.001^b^

^18^FDG PET, fluorodeoxyglucose positron emission tomography; CT, computed tomography; FDG+, fluorodeoxyglucose avidity; US, cervical ultrasound; IQR, interquartile range. An em dash (—) was noted when there were no patients with these characteristics.

^*^In case a patient had multiple suspicious lymph nodes, the largest was analyzed.

^a^ *P*-value was calculated using a two-sided χ ^2^ test.

^b^ *P*-value was calculated using Mann–Whitney *U* test.

### Diagnostic accuracy

Excluding patients with a suspicious cervical lymph node of unknown etiology, 103 patients had one or more suspicious lymph nodes, as shown in Table 2. Cervical ultrasound identified 97 suspicious cervical lymph nodes, of which 36 were true positive (malignant), 61 were false positive (benign), and two malignant cervical lymph nodes were missed, false negative. ^18^FDG PET-CT detected 53 suspicious cervical lymph nodes, of which 38 were true positive, 15 false positive, and zero false negative. For cervical ultrasound, this resulted in a sensitivity of 94.7% (95% CI: 82.3–99.4), a specificity of 91.3% (95% CI: 89.0–93.3), a PPV of 37.1% (95% CI: 31.5–43.1), and an NPV of 99.7% (95% CI: 98.8–99.9). For ^18^FDG PET-CT, this resulted in a sensitivity of 100% (95% CI: 90.8–100), a specificity of 97.9% (95% CI: 96.5–98.8), a PPV of 71.7% (95% CI: 60.6–80.7), and an NPV of 100% (95% CI: 99.5–100). Because ^18^FDG PET-CT showed no false negatives, McNemar’s test could not be performed. Diagnostic accuracy of the different modalities is displayed in Table 5.

**Table 5:** Diagnostic accuracy measures of ^18^FDG PET-CT and ultrasound for the detection of cervical lymph nodes

		^18^FDG PET-CT *N* = 738	US *N* = 738
		(95% CI)	(95% CI)
**Test validity**	Sensitivity	1.0 (0.91–1.0)	0.95 (0.82–0.99)
	Specificity	0.98 (0.96–0.98)	0.91 (0.89–0.93)
	PPV	0.71 (0.61–0.81)	0.37 (0.31–0.43)
	NPV	1.0 (0.99–1.0)	1.0 (0.99–1.0)
**Diagnostic accuracy**		0.98 (0.97–0.99)	0.91 (0.89–0.93)

^18^FDG PET, fluorodeoxyglucose-positron emission tomography; CT, computed tomography; US, cervical ultrasound; CI, confidence interval; PVV, positive predictive value; NPV, negative predictive value. Patients with an unknown etiology of lymph node were excluded.

### Change of management

All malignant lymph nodes proved to be metastatic disease from the esophageal primary tumor. For 34 of 38 patients (89.5%), the disease management plan changed after the discovery of a malignant cervical lymph node. For 19 (50.0%) patients, the treatment plan changed from curative to palliative intent. For 15 (39.5%) patients, the treatment plan changed from neoadjuvant chemoradiotherapy combined with surgery to definitive chemoradiotherapy.

## DISCUSSION

This study investigated the diagnostic accuracy of ultrasound over ^18^FDG PET-CT alone for the detection of suspicious cervical lymph nodes during the diagnostic workup for esophageal cancer. Cervical lymphadenopathy was detected in 112 (15%) patients. Of those, 33.9% were confirmed malignant through FNA. Malignant cervical lymph node involvement was significantly associated with squamous cell carcinoma, proximal tumor location, higher cN stage, and FDG avidity. Malignant nodes were also significantly larger and more often located at level IV compared to benign nodes. Diagnostic performance varied between modalities. Cervical ultrasound showed high sensitivity (94.7%) and NPV (99.7%) but a low PPV (37.1%), whereas ^18^FDG PET-CT demonstrated perfect sensitivity and NPV (both 100%) and a markedly higher PPV (71.7%).

As esophageal cancer patients with distant cervical lymph node metastases are considered to have stage IV disease, thorough and accurate detection of lymph node metastases is of great importance to determine treatment strategy and to prevent overtreatment. Diagnosing these lymph node metastases often changes the course of treatment, as in 89.5% of cases, the diagnosis of a malignant cervical node led to a change in treatment strategy. In 50.0% of patients, the plan shifted from curative to palliative intent. As analysis revealed a significantly higher incidence of squamous cell carcinoma in the malignant group, and tumor location also differed significantly between groups, with tumors in the distal esophagus or GEJ more often associated with benign cervical nodes, the use of cervical ultrasound may be considered in high-risk subgroups. However, no malignant cervical lymph nodes were missed on ^18^FDG PET-CT. Notably, the two patients with malignant cervical lymph nodes that were missed on cervical ultrasound but detected with ^18^FDG PET-CT both had a squamous cell carcinoma located in the mid-esophagus.

Current literature on the detection of cervical lymph nodes is conflicting and has only been performed in smaller cohorts. Goense *et al.* found a sensitivity of 85% and a specificity of 91% for the detection of lymph node metastases using ^18^FDG PET-CT,^7^ a lower sensitivity than 100% and a specificity of 97.9% found in this study. For ultrasound, they found a sensitivity of 73% and a specificity of 84%, also lower than the sensitivity of 94.7% and the specificity of 97.9% found in this study. They found that no additional malignant lymph nodes were detected with ultrasound. However, the study only included 163 patients with a cervical lymph metastasis incidence of 14%. A systematic review and meta-analysis included four studies comprising a total of 567 patients who underwent ^18^FDG PET-CT and ultrasound for the diagnostic workup of esophageal cancer.^6^ In one of the included studies, cervical ultrasonography detected additional cervical lymph node metastases in 4% (3/74) of patients over standalone ^18^FDG PET-CT. In the other three studies, ultrasound did not detect cervical lymph node metastases in addition to a negative finding on ^18^FDG PET-CT. In a large systematic review and meta-analysis from Leng *et al.* on the diagnostic accuracy of ultrasound for the detection of cervical lymph node metastasis in esophageal cancer patients, which included 22 studies comprising a total of 3.513 patients, found a sensitivity of 84% and a specificity of 93% for lymph nodes ≤5 mm and a sensitivity of 94% and a specificity of 98% for lymph nodes >5 mm.^18^ This is comparable to the overall sensitivity of 94.7% and specificity of 97.9% for ultrasound found in this study.

An explanation for the differences found in these studies is that imaging techniques tend to improve over time with increasing spatial resolution, and therefore, the diagnostic accuracy of ^18^FDG PET-CT might be of poorer quality in the earlier years. Currently, a newer Total Body ^18^FDG PET-CT is available, which has a higher spatial resolution and lower patient radiation dosage, which will further enhance diagnostic accuracy. Unfortunately, this scanner was not yet available during our inclusion period and is also not available in all hospitals. This study encompasses a relatively long retrospective period, during which notable advancements in ^18^FDG PET-CT technology likely occurred. Improvements in resolution may have influenced the accuracy outcomes in this study. This would likely have resulted in improved diagnostic accuracy in the later years. However, this trend was not reflected in the absolute numbers, and the sample size was too small to allow for meaningful statistical analysis.

An explanation for the observed differences in the performance of cervical ultrasound compared to ^18^FDG PET-CT, is that ultrasound has several inherent limitations that should be considered when interpreting the results. For instance, its relatively low positive predictive value indicated that benign cervical lymph nodes were more frequently misclassified as suspicious. A key weakness of ultrasound is its high operator dependency, which can lead to variability in diagnostic performance across institutions and examiners. Compared to ^18^FDG PET-CT, the accuracy of ultrasound is strongly influenced by the examiner’s experience, patient body habitus, and the anatomical accessibility of the lesion. Moreover, its sensitivity may be reduced for small, deep, or posteriorly located lesions, and distinguishing malignant from benign lymph nodes can be challenging when sonographic features overlap. One subgroup though, for which cervical ultrasound might be indicated, is patients with an ^18^FDG PET-CT negative primary tumor.

To our knowledge, this is the largest study to report on the diagnostic accuracy of ultrasound over ^18^FDG PET-CT for the detection of distant cervical lymph node metastases for esophageal cancer. A limitation of this study is that it is not known how many patients had a malignant cervical lymph node that was not detected by either ^18^FDG PET-CT or ultrasound. Fine needle aspiration was used as the gold standard. However, it would be impossible to conduct fine needle aspirations for all patients and all lymph nodes. Therefore, diagnostic accuracy could not be calculated based on these data. Known literature describes an overall diagnostic sensitivity, specificity, positive predictive value, and negative predictive value of fine needle aspiration of cervical lymph nodes to be 90.9%, 67.2%, 82.6%, and 81.3%, respectively.^19^ These values, however, were not specific to metastases from esophageal cancer. Unfortunately, we did not have data on disease recurrence. When malignant cervical lymph nodes were found after start of treatment, it was unclear if this was a missed cervical lymph node or a sign of disease progression. For nine patients, the etiology of the cervical lymph node remained unclear, partially because fine needle aspiration was not conducted for patients with disseminated disease elsewhere, as this was deemed clinically not relevant. Exclusion of these patients may have a selection bias, potentially leading to under- or overestimation of diagnostic accuracy. However, as a suspicious cervical lymph node is less clinically relevant in these patients, the reported outcomes in this study are more useful for application in the diagnostic workup population.

In conclusion, this study demonstrated that ultrasound has a poor PPV for the detection of cervical lymph node metastases of esophageal cancer, resulting in more fine needle aspirations being conducted for benign lesions. ^18^FDG PET-CT had perfect sensitivity and NPV and higher PPV, indicating that cervical ultrasound offers no additional diagnostic value over ^18^FDG PET-CT alone in the assessment of cervical lymph node metastases in esophageal cancer. Therefore, patients should undergo routine ^18^FDG PET-CT during the diagnostic workup and cervical ultrasound should only be performed on indication. Caution is warranted for patients with squamous cell carcinoma, proximal tumor location, and higher cN stage, as these factors were significantly associated with cervical lymph node metastasis.

## Notes

Declarations of interest: Mark I. van Berge Henegouwen is a consultant for Viatris, Johnson & Johnson, Stryker, Intuitive, BBraun and Medtronic. All fees paid to institution. Suzanne Gisbertz is a consultant for Medicaroid, J&J, Olympus. All fees paid to institution.

Financial support: This research received no specific grant from any funding agency in the public, commercial, or not-for-profit sectors.

Data availability: The data that support the findings of this study are not publicly available due to their containing information that could compromise the privacy of research participants but are available from one of the corresponding authors [J.R. van Doesburg or S.S. Gisbertz] upon reasonable request.

CRediT author Statement: Jasmijn Rose-Marie van Doesburg (Data curation, Formal analysis, Investigation, Methodology, Project administration, Visualization, Writing—original draft, Writing—review & editing, Involved in data curation), Nannet Schuring (Conceptualization, Data curation, Investigation, Methodology, Writing—review & editing, Involved in the conceptualization of the study, Involved in data curation, Contributed to the interpretation of the results, reviewed and edited the manuscript), Mark HM Vries (Data curation, Writing—review & editing, Involved in the conceptualization of the study, Involved in data curation, Contributed to the interpretation of the results, reviewed and edited the manuscript), Pim de Graaf (Conceptualization, Data curation, Writing—review & editing, Involved in the conceptualization of the study, Involved in data curation, Contributed to the interpretation of the results, reviewed and edited the manuscript), Katya Duvivier (Data curation, Writing—review & editing, Involved in data curation, Contributed to the interpretation of the results, reviewed and edited the manuscript), F Daams (Supervision, Writing—review & editing, Contributed to the interpretation of the results, reviewed and edited the manuscript), Mark van Berge Henegouwen (Conceptualization, Data curation, Investigation, Methodology, Supervision, Writing—review & editing, Involved in the conceptualization of the study, Contributed to the interpretation of the results, reviewed and edited the manuscript), and Suzanne Sarah Gisbertz (Conceptualization, Investigation, Methodology, Writing—review & editing, Involved in the conceptualization of the study, Contributed to the interpretation of the results, reviewed and edited the manuscript), N. Schuring, M.H.M. Vries, P. de Graaf, S.S. Gisbertz and M.I. van Berge Henegouwen. Project administration was done by N. Schuring and J.R. van Doesburg. The study was supervised by S.S. Gisbertz; J.R. van Doesburg wrote the original draft

## References

[1] Shapiro J, van Lanschot J J B, Hulshof M C C M et al. Neoadjuvant chemoradiotherapy plus surgery versus surgery alone for oesophageal or junctional cancer (CROSS): long-term results of a randomised controlled trial. Lancet Oncol 2015; 16(9): 1090–8. 10.1016/S1470-2045(15)00040-6. 26254683

[2] Sobin L H, Gospodarowicz M K, Wittekind C. TNM classification of malignant tumours. John Wiley & Sons, New York, NY, 2011.

[3] Amin M B et al. AJCC cancer staging manual, 8th edn. Springer International Publishing, New York, NY, 2018.

[4] Integraal Kankercentrum Nederland (IKNL) . Landelijke richtlijn: Oesofaguscarcinoom, vol. Versie 3.1. Utrecht, The Netherlands: Integraal Kankercentrum Nederland (IKNL).

[5] Nederlandse Vereniging van Maag-Darm-Leverartsen . (2015) *Dutch national guidelines esophageal cancer.* Oncoline version 3.1. oncoline.nl/oesofaguscarcinoom.

[6] Goense L, Meziani J, van Rossum P S N et al. Limited additional value of cervical ultrasonography over a negative 18F-FDG PET/CT for diagnosing cervical lymph node metastases in patients with esophageal cancer: a systematic review and meta-analysis. Nucl Med Commun 2018; 39(7): 645–51. 10.1097/MNM.0000000000000847. 29672463

[7] Goense L, Meziani J, van Rossum P S N et al. Cervical ultrasonography has no additional value over negative 18F-FDG PET/CT scans for diagnosing cervical lymph node metastases in patients with oesophageal cancer. Eur Radiol 2018; 28(5): 2031–7. 10.1007/s00330-017-5136-x. 29218619 PMC5882618

[8] Li B, Li N, Liu S et al. Does [18F] fluorodeoxyglucose–positron emission tomography/computed tomography have a role in cervical nodal staging for esophageal squamous cell carcinoma? J Thorac Cardiovasc Surg 2020; 160(2): 544–50. 10.1016/j.jtcvs.2019.11.046. 31932053

[9] Omloo J M T, van Heijl M, Smits N J et al. Additional value of external ultrasonography of the neck after CT and PET scanning in the preoperative assessment of patients with esophageal cancer. Dig Surg 2009; 26(1): 43–9. 10.1159/000193630. 19155627

[10] Schreurs L M A, Verhoef C C P M, van der Jagt E J, van Dam G M, Groen H, Plukker J T M. Current relevance of cervical ultrasonography in staging cancer of the esophagus and gastroesophageal junction. Eur J Radiol 2008; 67(1): 105–11. 10.1016/j.ejrad.2007.06.022. 17681735

[11] Blom R L G M, Vliegen R F A, Schreurs W M J et al. External ultrasonography of the neck does not add diagnostic value to integrated positron emission tomography–computed tomography (PET–CT) scanning in the diagnosis of cervical lymph node metastases in patients with esophageal carcinoma. Dis Esophagus 2012; 25(6): 555–9. 10.1111/j.1442-2050.2011.01289.x. 22150869

[12] von Elm E, Altman D G, Egger M, Pocock S J, Gøtzsche P C, Vandenbroucke J P. The STROBE statement: guidelines for reporting observational studies. Int J Surg 2014; 12(12): 1495–9. 10.1016/j.ijsu.2014.07.013. 25046131

[13] Cohen J F, Korevaar D A, Altman D G et al. STARD 2015 guidelines for reporting diagnostic accuracy studies: explanation and elaboration. BMJ Open 2016; 6(11): e012799. 10.1136/bmjopen-2016-012799. PMC5128957 28137831

[14] van Hagen P, Hulshof M C C M, van Lanschot J J B et al. Preoperative chemoradiotherapy for esophageal or junctional cancer. N Engl J Med 2012; 366(22): 2074–84. 10.1056/NEJMoa1112088. 22646630

[15] Prativadi R, Dahiya N, Kamaya A, Bhatt S. Chapter 5 ultrasound characteristics of benign vs malignant cervical lymph nodes. Semin Ultrasound CT MR 2017; 38(5): 506–15. 10.1053/j.sult.2017.05.005. 29031367

[16] Ying M, Ahuja A. Sonography of neck lymph nodes. Part I: normal lymph nodes. Clin Radiol 2003; 58(5): 351–8. 10.1016/S0009-9260(02)00584-6. 12727162

[17] Ahuja A, Ying M. Sonography of neck lymph nodes. Part II: abnormal lymph nodes. Clin Radiol 2003; 58(5): 359–66. 10.1016/S0009-9260(02)00585-8. 12727163

[18] Leng X F, Zhu Y, Wang G P, Jin J, Xian L, Zhang Y H. Accuracy of ultrasound for the diagnosis of cervical lymph node metastasis in esophageal cancer: a systematic review and meta-analysis. J Thorac Dis 2016; 8(8): 2146–57. 10.21037/jtd.2016.07.71. 27621871 PMC4999759

[19] Hafez N H, Tahoun N S. Reliability of fine needle aspiration cytology (FNAC) as a diagnostic tool in cases of cervical lymphadenopathy. J Egypt Natl Canc Inst 2011; 23(3): 105–14. 10.1016/j.jnci.2011.09.009. 22776815

